# Nutrient uptake of two microalgae from culture medium under nickel treatment

**DOI:** 10.55730/1300-0152.2805

**Published:** 2026-05-13

**Authors:** Dilara ERDAĞI, Büşranur ŞEKER, Celal CANER, Gizem ÇETİN, Hüseyin ALTUNDAĞ, Hatice TUNCA

**Affiliations:** 1 Department of Biology, Faculty of Science, Sakarya University, Sakarya, Turkiye; 2 Department of Chemistry, Faculty of Science, Sakarya University, Sakarya, Turkiye

**Keywords:** Bioremediation, growth parameters, metal removal, nutrient uptake, microalgae

## Abstract

**Background/aim:**

Phycoremediation is essential for purifying wastewater of heavy metals to ensure a sustainable environment, as these components can be detrimental to organisms upon entering the food chain. Among heavy metals, nickel is vital at low concentrations due to its presence in the active sites of many metalloenzymes in algae. However, at high concentrations, it disrupts the functioning of many metabolic reactions. This study examines the effects of nickel treatment on the uptake of macro- and micronutrients in medium by algae via ion exchange pathways or in competition with other metals. The study aims to determine the effects of nickel exposure on macro- and micronutrient uptake in algae and evaluate the bioremediation potential of *Desmodesmus pannonicus* and *Scenedesmus aldavei* under varying NiSO_4_ concentrations. Additionally, it assesses growth responses, elemental composition changes, and alterations in functional groups to determine the tolerance and effectiveness of these algae in Ni(II) removal from contaminated environments.

**Materials and methods:**

The bioremediation abilities of *Desmodesmus pannonicus* and *Scenedesmus aldavei* were evaluated spectrophotometrically by measuring growth rate, chlorophyll-*a*, and chlorophyll-*b* for 14 days. Bioremediation properties were also analyzed using inductively coupled plasma optical emission spectroscopy.

**Results:**

Growth parameters varied with concentrations (p ≤ 0.05). Both species were found to have bioremediation capabilities up to the concentration of 1 M NiSO_4_, which increased the absorption of Na^+^ ion content (p ≤ 0.05). However, the Mg(II), Ca(II), and K contents in the dry mass changed with the concentration (p ≤ 0.05). FTIR analysis was used to identify changes in specific functional groups in the samples, including carboxyl, amine, hydroxyl, and carbonyl.

**Conclusion:**

*Desmodesmus pannonicus* and *Scenedesmus aldavei* exhibit significant potential for Ni(II) bioremediation, showing tolerance to NiSO_4_ concentrations of up to 1 M. Compared to other organisms in the literature, *Scenedesmus aldavei* is an excellent model for phycoremediation studies.

## 1. Introduction

Phycoremediation, which is essential for a sustainable environment, is used to purify wastewater of organic and inorganic pollutants, such as heavy metals, nutrients, pesticides, and xenobiotics, using algae (Kaloudas et al., 2021; Priyadharshini et al., 2021; Ankit et al., 2022). This biorefining process, which does not cause secondary contamination, is increasingly favored over other methods because it yields valuable biomass for biofuel production and other industrial applications (Mathimani and Pugazhendhi, 2019; Kandasamy et al., 2021). The adsorption and absorption of metals as physiological responses of microalgae affect the success of these applications (Mehariya et al., 2024). Rapid adsorption processes, independent of metabolism, involve accumulation on the cell membrane and are unaffected by light, heat, and other metabolic inhibitors (Lv et al., 2023; Mahlangu et al., 2024). In contrast, absorption is a metabolism-dependent process that occurs more slowly and can be affected by metal accumulation. Metals may accumulate in different cell compartments during this process (Leong and Chang, 2020; Huang et al., 2021). Since the adsorption capacity of algae depends on negatively charged functional groups on the cell surface, the charge of the metal and the reactivity of other metal ions in solution affect the binding activity (Nasir et al., 2019; Pradhan et al., 2019; Spain et al., 2021). The presence of multiple metal ions in industrial, medical, and domestic wastewater, as well as culture media, complicates environmental and biotechnological applications, requiring comprehensive analysis (Nasir et al., 2019; Ramírez-García et al., 2019).

There are limited studies in the literature on how metal uptake affects nutrient uptake in algae. Tüzün et al. (2005) evaluated the biosorption capacity of *Chlamydomonas reinhardtii* biomass for mixtures containing Hg(II), Cd(II), and Pb(II) ions. They found decreased uptake in mixed solutions compared to a remediation study performed with metals individually. The observed decrease in the biosorption of Hg(II) and Cd(II) ions was more pronounced compared to Pb(II) ions, whereas algal biomass had the highest biosorption capacity for Pb(II) ions. Senthilkumar et al. (2006) investigated how cationic metal ions such as Na^+^, K^+^, Ca(II), and Mg(II), which are commonly found in industrial wastewater, affected the zinc uptake capacity of *Ulva reticulata*. They observed that increasing concentrations of Ca(II) and Mg(II) together reduced zinc uptake, while K^+^ and Na^+^ had minimal competition for binding sites. Saavedra et al. (2018) published a comparative remediation study of toxic elements including arsenic, boron, copper, manganese, and zinc in monometallic and multimetallic solutions using the following green microalgae species: *Chlamydomonas reinhardtii*, *Chlorella vulgaris*, *Scenedesmus almeriensis*, and native *Chlorophyceae* spp. They reported decreased boron removal in multimetallic solutions, except for *Chlorophyceae* spp. Tunali and Yenigun (2021) analyzed the remediation properties of multimetal mixtures of Ag^+^ and Nd^3+^ compounds from wastewater in living and dried cells of *Chlorella vulgaris*. They found that multimetal solutions reduced uptake compared to single-metal biosorption.

Among heavy metals, which are primary sources of aquatic pollution, nickel is a significant contaminant at high concentrations in aquatic environments due to mining research and its use in various industries (Boyle and Robinson, 2024). Reck et al. (2008) reported that nickel could reach concentrations of up to 3 mM in highly affected aquatic resources. Excessive nickel accumulation within the cell can disrupt subcellular structures and induce toxicity. This metal is suitable for phycoremediation due to its ability to bind to anionic groups on cell membranes and its increasing contamination of aquatic environments. Klimmek et al. (2001) studied the biosorption of nickel for the algal species *Lyngbya taylorii*. Sousa et al. (2018) evaluated the toxicity of nickel for *Pseudokirchneriella subcapitata* and found that increased levels of reactive oxygen species led to disruptions in algal morphology and the cell cycle. García-García et al. (2018) examined the physiological effects of nickel accumulation on *Euglena gracilis* and observed changes in intracellular levels of macromolecules in response to treatments. Santos et al. (2019) evaluated nickel interaction with other metals and its effects on nutrient uptake for *C. vulgaris*. These studies on nickel were conducted with different algae than those used in the present study and there is no information in the literature on the phycoremediation abilities of the algae used in the present study.

*Desmodesmus pannonicus* and *Scenedesmus aldavei* have been used in various biotechnological applications such as carbon emission reduction, bioremediation, and lipid-based biofuel production (Allard and Templier, 2000; Balouch et al., 2023; Kumar et al., 2024). However, the nickel uptake of these algae, which may be essential biosorbents for phycoremediation, and their effects on this metal remain unexplored. In this regard, the present study makes a novel contribution to the literature. This study hypothesizes that i) the algae *Desmodesmus pannonicus* and *Scenedesmus aldavei*, which contain important anionic compounds such as carboxyl, carbonyl, hydroxyl, and sulfhydryl in their cell walls, have a high capacity to absorb cationic Ni(II) metal and that ii) Ni(II) content acts as an activator or inhibitor for the uptake of macronutrients and other micronutrients in the medium. Accordingly, this study aimed to evaluate the Ni(II) biosorption performances of *Desmodesmus pannonicus* and *Scenedesmus aldavei* and to investigate how nickel exposure influences their growth behavior, photosynthetic pigment dynamics, and uptake of essential macro- and micronutrients in order to clarify their potential applicability in phycoremediation processes.

## 2. Materials and methods

### 2.1. Culture conditions

The microalgae strains *Desmodesmus pannonicus* SAU001 (isolated from Sakarya, Türkiye) and *Scenedesmus aldavei* SAU002 (isolated from Trabzon, Türkiye) were cultured in the Algal Physiology Laboratory of the Sakarya University Faculty of Science to evaluate nickel uptake capability. Enriched BG11 broth (Rippka, 1981) was used for the culture medium and the volume was gradually increased over time. The single-culture agar method was used for isolation (Gerloff et al., 1950). The guidelines of Huber-Pestalozzi (1983) were used for species identification. Both strains were incubated under autotrophic conditions in BG11 medium (pH 7.5; Rippka, 1981) at 5000 lx with a 12:12-h light/dark cycle at 27 °C until a culture volume of 500 mL was reached. Cultures were renewed at McFarland turbidity of 0.9–1 and Ni(II)-NiSO_4_ (0.2, 0.4, 0.6, 0.8, and 1 M) was added to the culture medium at a final concentration for each treatment. The applied Ni(II) concentrations were selected based on environmental concentrations derived from urban stormwater samples (Begum et al., 2022). A volume of 3 mL per sample was used for cell growth parameters and 20 mL per sample was used for analytical analysis.

### 2.2. Cell growth and pigment analysis

Optical density (OD_750_), chlorophyll-*a*, and chlorophyll-*b* levels were measured every 24 h using a BioTek Epoch microplate reader (Agilent Technologies, Santa Clara, CA, USA) to determine the growth rate, which revealed the greatest growth in the exponential phase by the 14th day. The extracts were obtained according to Rippka (1981). Samples diluted at 1/10 in methanol were vortexed for 1 min and then incubated in the dark for 20 min. Dilutions were centrifuged at 42,582 × *g* for 2 min and supernatants were measured at 652, 665, and 470 nm using a spectrophotometer. Chlorophyll-*a* (c_a_) and chlorophyll-*b* (c_b_) values were calculated according to Ritchie (2008) as seen in Eqs. (1) and (2). Cellular growth was measured every 24 h throughout the growth phase using fresh optical density readings to determine the correlation between Ni(II) removal as described in subsection 2.3. All experiments related to cell growth parameters were performed in quadruplicate and data were expressed as mean ± standard error. Half maximal effective concentration (EC_50_) values were calculated on the fifth day using Origin 8.5 software (OriginLab Corporation, Northampton, MA, USA) and the growth model.


(1) $${\text{c}}_{\text{a}}\;(\mu \text{g}/\text{mL})=16.82\;{\text{A}}_{665.2}-9.28{\text{A}}_{652.4}$$

(2) $${\text{c}}_{\text{b}}\;(\mu \text{g}/\text{mL})=36.92\;{\text{A}}_{652.4}-16.54{\text{A}}_{665.2}$$

### 2.3. Analytical analysis

Analytical analysis was conducted using a method modified based on that of Tambat et al. (2023). The adsorption of nickel metal compounds and other metal ions was evaluated in direct relation to dried microalgae biomass with incubation in an oven. Maximum microbial dried biomass was obtained on the 14th day, coinciding with the logarithmic growth phase. At this point, 20 mL of broth culture was collected and centrifuged at 2822 × *g* for 10 min (NF200 centrifuge, Nüve, Ankara, Türkiye). The supernatant and the microalgae–dried biomass pellets were carefully separated into premeasured tubes. After the pellets were dried, 5 mL of aqua regia was added to the dried biomass. Both samples (*D. pannonicus* and *S. aldavei*) derived from the pellets were filtered through 0.22-μm filter paper. Sample analysis was performed with a Spectro Arcos FHE-16 Inductively Coupled Plasma Optical Emission Spectrometer (Spectro Analytical Instruments, Kleve, Germany). Before the analysis, the sensitivity and stability of the instrument were optimized to an analytical range of 8 ppm. Samples derived from the pellets were diluted 20-fold from their initial concentrations for metal ion analysis, while supernatants were measured directly. Nickel concentrations were calculated from estimated nickel sulfate concentrations. The uptake levels of Ni(II) and other nutrients were calculated from adsorbed fresh cultures and supernatants using ICP-OES. All experiments, including the steps of the analytical analysis, were performed with three biological and two technical replicates, and data were expressed as mean ± standard error.

### 2.4. FTIR spectroscopy

Fourier transform infrared (FTIR) spectroscopic analysis was conducted according to a method modified based on that of Tambat et al. (2023) using an IRAffinity-1S FT-IR device (Shimadzu, Kyoto, Japan). The pellets were prepared with a small amount of Ni-adsorbed cultures of *Desmodesmus pannonicus* SAU001 and *Scenedesmus aldavei* SAU002. At the end of the 14th day, 30 mL of broth culture was collected and centrifuged for 10 min at 2822 × *g*. The dried biomass was carefully crushed with mortars and pestles and mixed with KBr at a ratio of 1:20. The analysis was carried out in the frequency range of 400–4000 cm^−1^ for transmission spectra.

### 2.5. Statistical analysis

The experimental design aimed to minimize assay errors and variability by ensuring replicates for each procedure. Statistical significance between mean values was assessed using analysis of variance (ANOVA) with the Tukey post hoc test in IBM SPSS 20.0 for Windows (IBM Corp., Armonk, NY, USA) at a 95% confidence level. Two-way ANOVA was employed to analyze growth parameters in a time- and dose-dependent manner, while one-way ANOVA was applied for bioremediation studies. Graphical presentations include standard errors, with significant differences marked by asterisks.

## 3. Results and discussion

### 3.1. Effects of nickel treatment on growth parameters

OD_750_ absorbance and chlorophyll-*a* and chlorophyll-*b* pigment levels were monitored to determine the growth dynamics of *Desmodesmus pannonicus* and *Scenedesmus aldavei* under NiSO_4_ treatment over 14 days. ANOVA Tukey test results indicated that cell density increased over time in both species. However, the effect of nickel exposure differed significantly between the species.

**Figure 1:**
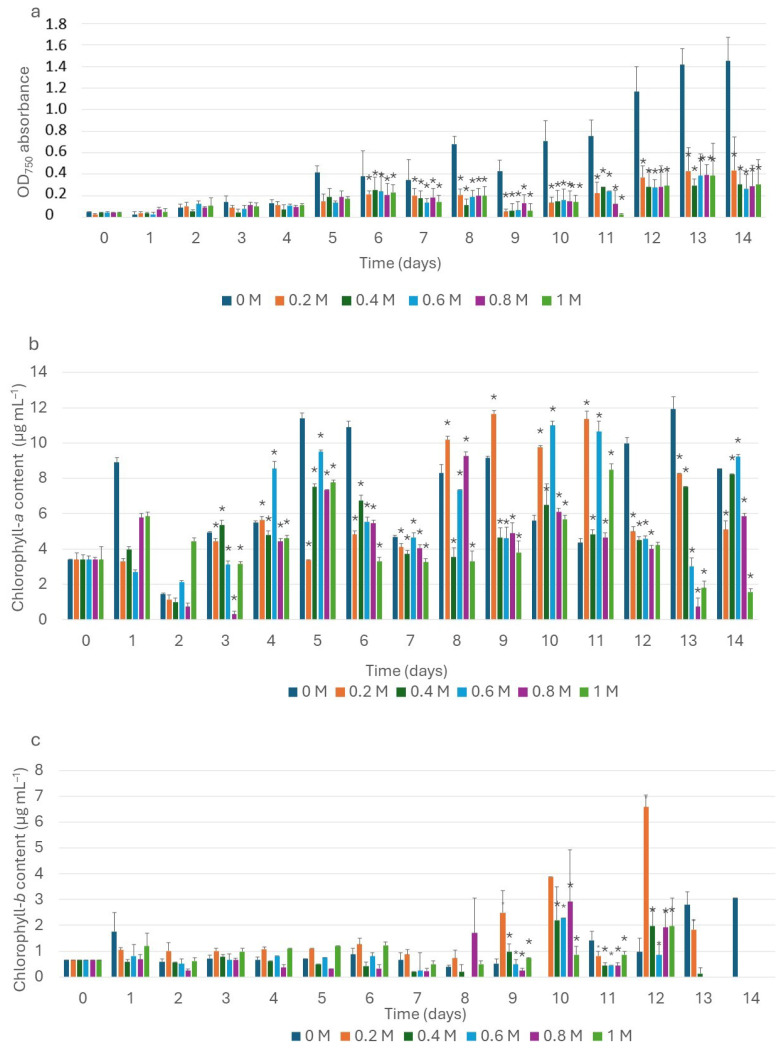
**(a)** OD_750_ absorbance, **(b)** chlorophyll-*a*, and **(c)** chlorophyll-*b* pigments of *Scenedesmus aldavei* under NiSO_4_ toxicity for 14 days.

The fact that OD_750_ values in *Scenedesmus aldavei* showed changes, particularly after day 6, suggests that a tipping point was reached in the growth rate due to the effect of nickel. The significant decrease in chlorophyll-*b* levels compared to the control group at all concentrations starting from day 8 (p ≤ 0.05) indicates that the photosynthetic apparatus was suppressed, particularly at the level of accessory pigments. In contrast, the emergence of a significant difference in chlorophyll-*a* levels was only seen on the 14th day, which indicates that this species can maintain its primary photosynthetic pigment for a longer period and thus exhibit a more tolerant response to stress (Figure 1).

**Figure 2:**
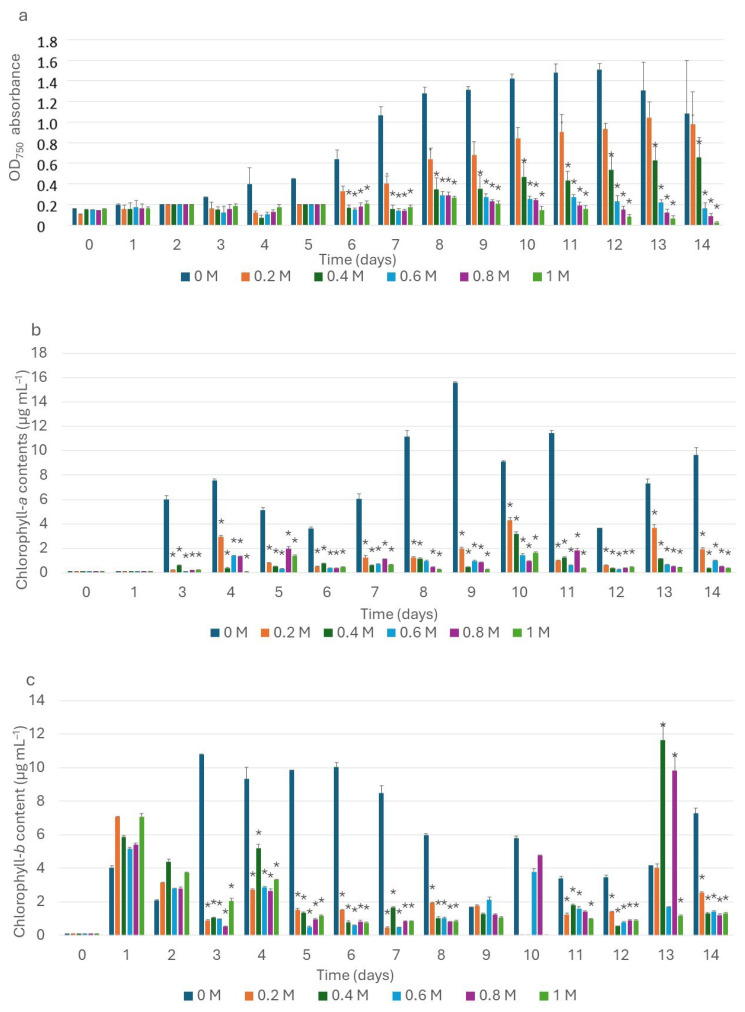
**(a)** OD_750_ absorbance, **(b)** chlorophyll-*a*, and **(c)** chlorophyll-*b* pigments of *Desmodesmus pannonicus* under NiSO_4_ toxicity for 14 days.

In *Desmodesmus pannonicus*, it was observed that NiSO_4_ concentrations in the range of 0.2–1 M caused significant changes (p ≤ 0.05) in the OD_750_ growth curves compared to the control group starting from day 7. Although the fact that chlorophyll-*a* levels in this species were higher than those of the control at all concentrations starting from the third day might suggest an initial stress adaptation response, the significant decrease in chlorophyll-*b* levels after the fourth day (p ≤ 0.05; Figure 2) indicates that the photosynthetic system was generally suppressed. Overall, Ni(II) application caused a net decrease in chlorophyll content in *Desmodesmus pannonicus*, resulting in a more sensitive physiological response.

The observed decrease in OD_750_ values of *Scenedesmus aldavei* accompanied by relatively higher or fluctuating chlorophyll-*a* levels suggests a decoupling of total biomass accumulation and functional pigment content under Ni(II) stress (Figure 1). OD_750_ reflects overall culture density and light scattering caused by intact cells; therefore, its reduction indicates inhibition of cell proliferation, increased cell lysis, or changes in cell morphology and aggregation induced by nickel toxicity (Stein, 1973; Barsanti and Gualtieri, 2022). In contrast, chlorophyll-*a* and chlorophyll-*b* dynamics are governed by both synthesis and stress-induced metabolic regulation, and they may not follow the same trend as cell density. This may also lead to inconsistencies among growth parameters.

The increase or fluctuation in chlorophyll-*a* under certain Ni concentrations can be attributed to a stress-induced acclimation response. Microalgae often activate protective mechanisms under moderate heavy metal stress, including the upregulation of chlorophyll-*a*-associated photosystem repair systems or the relative accumulation of chlorophyll-*a* due to the faster degradation of accessory pigments such as chlorophyll-*b*. This leads to an apparent increase or stabilization of chlorophyll-*a* even when growth is inhibited. Chlorophyll-*b* is generally more sensitive to environmental stress because it is primarily associated with light-harvesting antenna complexes, which are among the first targets of heavy metal-induced oxidative damage (Pinto et al., 2003).

The divergence between OD_750_ and chlorophyll profiles, as well as the intrapigment variability between chlorophyll-*a* and chlorophyll-*b*, thus indicates that Ni(II) exposure induces a complex stress response involving growth inhibition, pigment remodeling, and adaptive photosynthetic regulation rather than a simple linear suppression of algal physiology.

The EC_50_ values quantitatively support these results. Based on OD_750_ data, the EC_50_ value for NiSO_4_ was calculated as 3.76 mM (0.58 mg mL^−1^) for *Desmodesmus pannonicus* and 53.55 mM (8.29 mg mL^−1^) for *Scenedesmus aldavei*. These results indicate that *Scenedesmus aldavei* is significantly more resistant to nickel stress and is therefore a more suitable candidate for phytoremediation applications.

While the findings from the present study are generally consistent with the literature demonstrating the inhibitory effects of nickel toxicity on growth and photosynthetic pigments in microalgae, they highlight interspecies differences in tolerance more clearly. The EC_50_ values calculated in this study (3.76 mM for *Desmodesmus pannonicus* and 53.55 mM for *Scenedesmus aldavei*) indicate that *S. aldavei* exhibits a notably high resistance to nickel stress. Macoustra et al. (2021) investigated nickel toxicity in a *Chlorella* species in the presence of three different dissolved organic matter fractions and reported an EC_50_ value of 120 μg Ni L^−1^. In contrast, the values of 1.85 ± 0.17 mg L^−1^ reported by Guo et al. (2022) for *Phaeodactylum tricornutum* and 15 mg L^−1^ reported by El-Agawany and Kaamoush (2022) for *Dunaliella tertiolecta* indicate that these species exhibit lower tolerance to nickel stress. McKnight et al. (2023) also reported that *Symbiodinium* sp. showed low sensitivity to nickel exposure (EC_50_: >1600 μg Ni L^−1^). In addition, they reported EC_50_ values of 170 and 440 μg Ni L^−1^ for *Chlorella* sp. and *Monoraphidium arcuatum*, respectively. Similarly, the very low EC_50_ value of 0.121 mg L^−1^ reported by Bucková et al. (2023) for *Desmodesmus subspicatus* indicates that significant differences in sensitivity may exist even within the genus *Desmodesmus*. Furthermore, the observation by Dos Reis et al. (2024) of significant decreases in cell density and chlorophyll-*a* fluorescence in *Raphidocelis subcapitata* even at a Ni(II) concentration of only 0.35 mg L^−1^ supports nickel’s potent inhibitory effect on the photosynthetic apparatus. In this context, the tolerance profile of *S. aldavei*, characterized by the longer-lasting maintenance of chlorophyll-*a* levels and a high EC_50_ value, clearly demonstrates that this species is more resistant compared to many other microalgal species reported in the literature.

These comparisons confirm that nickel toxicity generally inhibits growth and photosynthetic pigments in microalgae and support the results of the present study. The data obtained here not only confirm the general effects of nickel toxicity but also strongly support the idea that *S. aldavei* could be a more advantageous candidate for phytoremediation applications compared to species previously highlighted in the literature. In particular, *Scenedesmus aldavei* has a high EC_50_ value and chlorophyll-*a* stability, indicating that this species possesses a physiology that is more resistant to heavy metal stress. In this context, it can be considered one of the leading species for phytoremediation after *Dunaliella tertiolecta*, as previously described in the literature (El-Agawany and Kaamoush, 2022).

### 3.2. Nickel bioremediation and FTIR analysis

**Figure 3:**
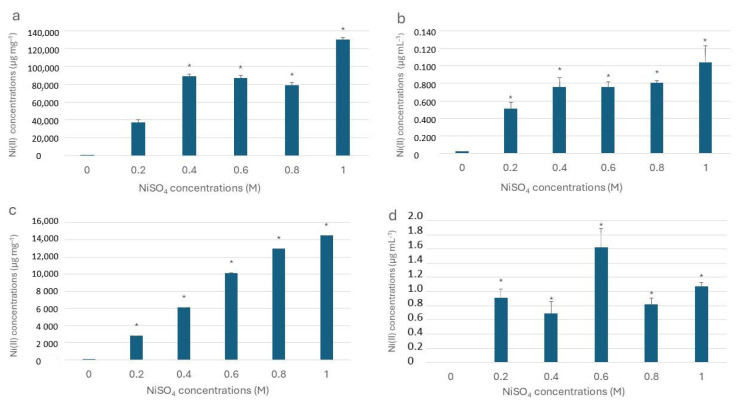
Bioremediation of Ni(II) in **(a)** sample of *Scenedesmus aldavei* dried biomass, **(b)** sample of *Scenedesmus aldavei* supernatant, **(c)** sample of *Desmodesmus pannonicus* dried biomass, and **(d)** sample of *Desmodesmus pannonicus* supernatant under NiSO_4_ toxicity.

As seen in Figure 3, the supernatant and biomass samples collected on day 14 clearly demonstrate the Ni(II) biosorption capacity of *Desmodesmus pannonicus* and *Scenedesmus aldavei*. The results of the ANOVA Tukey test (p ≤ 0.05) indicate that the Ni(II) removal capacity of both algal species increased significantly with increasing concentrations. This suggests that heavy metal uptake exhibits a dose-dependent response and that the interaction between the cell surface and the metal becomes more effective as the concentration increases.

When the present findings are evaluated alongside previous studies, it is evident that the Ni(II) biosorption capacity of microalgae varies considerably depending on species-specific characteristics as well as environmental and experimental conditions. Early research by Çetinkaya Dönmez et al. (1999) demonstrated that dried biomass of *Chlorella vulgaris*, *Scenedesmus obliquus*, and *Synechocystis* sp. effectively removed Ni(II) from aqueous solutions, with metal uptake increasing proportionally to the initial nickel concentration and reaching maximum levels at 250 mg L^−1^. Similarly, Wong et al. (2000) reported pronounced interspecific differences in nickel removal performance between *Chlorella vulgaris* and *Chlorella miniata*, showing that *C. miniata* removed more than 99% of Ni^2+^, whereas *C. vulgaris* exhibited only 33%–41% removal efficiency. The same study also revealed marked differences in uptake capacity, with maximum biosorption values of 641.76 μg g^−1^ for *C. vulgaris* and 1367.62 μg g^−1^ for *C. miniata*.

Environmental parameters were also shown to influence biosorption efficiency strongly. Aksu (2002) demonstrated that *Chlorella vulgaris* exhibited its highest Ni(II) biosorption capacity at 45 °C, pH 4.5, and initial nickel concentration of 250 mg L^−1^, with uptake capacity increasing from 48.1 to 60.2 mg g^−1^ with increasing temperature. More recent studies have generally reported moderate nickel uptake capacities for different microalgal taxa. Ahmad et al. (2020) found that *Phacus* sp. exhibited Ni(II) biosorption capacity of 8.82 ± 0.16 mg g^−1^, while Şen et al. (2022) reported maximum uptake capacity of 11.8 mg g^−1^ for *Cyanobacterium aponinum* at a Ni(II) concentration of 14.6 mg L^−1^. Likewise, Filová et al. (2021) reported relatively low metal uptake levels in *Raphidocelis subcapitata*, ranging from 0.023 to 0.496 μg mg^−1^ at different Ni(II) concentrations. Bucková et al. (2023) also demonstrated gradual nickel accumulation in *Desmodesmus subspicatus*, with intracellular metal levels increasing from 0.039 to 0.068 pg cell^−1^ between the second and fourth days of exposure to 80 μg L^−1^ Ni(II). Collectively, these studies indicate that nickel biosorption and accumulation efficiency differ substantially among algal species and are strongly affected by exposure concentration, biomass characteristics, and cultivation conditions.

In the present study, fresh *Desmodesmus pannonicus* biomass exhibited Ni(II) biosorption capacity of 14.52 ± 0.40 mg g^−1^ at 1 M NiSO_4_. In contrast, *Scenedesmus aldavei* showed a markedly higher uptake capacity of 130.03 ± 2.14 mg g^−1^ under the same conditions (p ≤ 0.05). Compared to the majority of previously reported microalgal biosorption capacities, the uptake performance of *S. aldavei* was substantially higher, suggesting that this species possesses exceptional tolerance and biosorption potential for nickel removal. These findings highlight substantial interspecific variability in Ni(II) biosorption and suggest that *S. aldavei* may be a promising candidate for future nickel bioremediation applications.

This high biosorption capacity suggests that, in addition to the potential of functional groups on the cell surface (such as carboxyl, hydroxyl, and phosphate groups) to bind metal ions, the cell wall structure and biomass properties also play significant roles. Furthermore, the use of living cells demonstrates that not only passive adsorption but also metabolic processes contribute to metal uptake (Volesky, 2001; Gadd, 2009; Fomina and Gadd, 2014).

**Figure 4:**
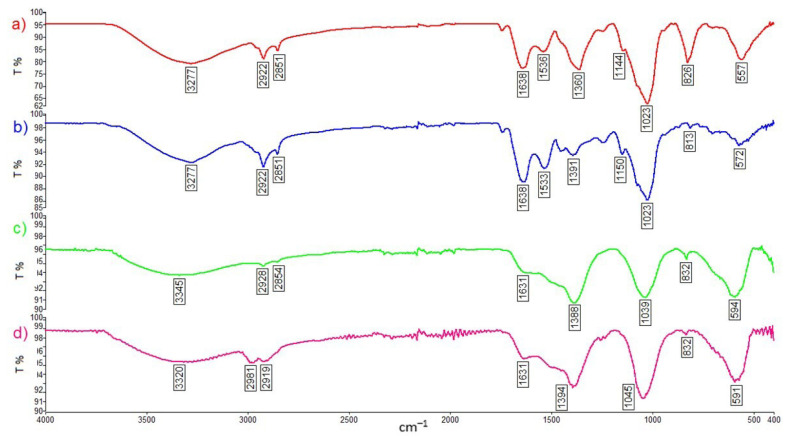
FTIR spectra of a) *Desmodesmus pannonicus* control, b) *Scenedesmus aldavei* control, c) *Desmodesmus pannonicus* under NiSO_4_ treatment, and d) *Scenedesmus aldavei* under NiSO_4_ treatment.

FTIR analysis was utilized to identify specific functional groups such as carboxyl, amine, hydroxyl, and carbonyl in the samples (Table). Examining the spectra, the peak intensity between 3030 and 3700 cm^−1^ corresponds to the stretching vibrations of O-H and N-H bonds, respectively (Waqar et al., 2023). The fingerprint region of the distinctive spectra for *Scenedesmus aldavei* and *Desmodesmus pannonicus* is presented in Figure 4. Peaks in the 2820–3030 cm^−1^ range represent −CH_3_ asymmetric and −CH_3_ symmetric stretching vibrations (Moraes et al., 2021). Peaks between 1579 and 1715 cm^−1^ indicate the presence of the amide I protein band and C=O stretching vibrations, while the 1484–1580 cm^−1^ range indicates the presence of the amide II protein band. Peaks between 1271 and 1484 cm^−1^ belong to the amide III protein band (Wei et al., 2020). The 1132–1197 cm^−1^ range confirms the presence of −COH groups in polysaccharides and P=O stretching vibrations of nucleic acids. Peaks between 980 and 1130 cm^−1^ further confirm the presence of C-O-C and C-OH groups in polysaccharides. Finally, the peak intensity between 400 and 950 cm^−1^ corresponds to the peaks of the C-H group outside the vibrational plane (Ferraro et al., 2021; Jacinto et al., 2023; Singh et al., 2023).

Overall, this study demonstrates that *Scenedesmus aldavei* in particular exhibits high performance in Ni(II) removal and that this species may be a strong candidate for the biological remediation of heavy metal contamination. The high values obtained, when compared to the literature, further highlight the potential of this species for environmental bioremediation applications.

### 3.3. Effects of nickel treatment on nutrient uptake mechanisms

**Figure 5:**
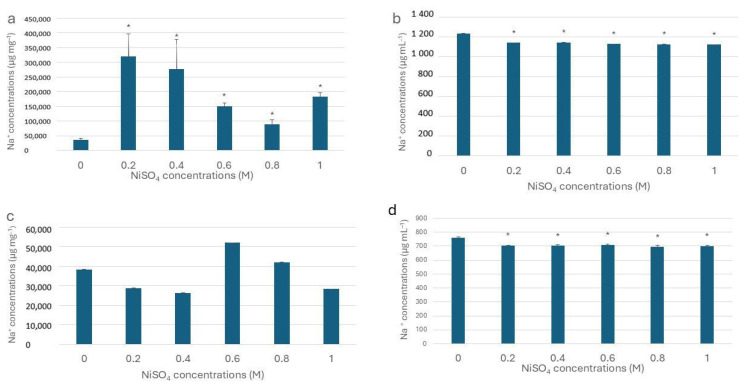
Uptake of Na^+^ ions in **(a)** sample of *Scenedesmus aldavei* dried biomass, **(b)** sample of *Scenedesmus aldavei* supernatant, **(c)** sample of *Desmodesmus pannonicus* dried biomass, and **(d)** sample of *Desmodesmus pannonicus* supernatant under NiSO_4_ toxicity.

**Figure 6:**
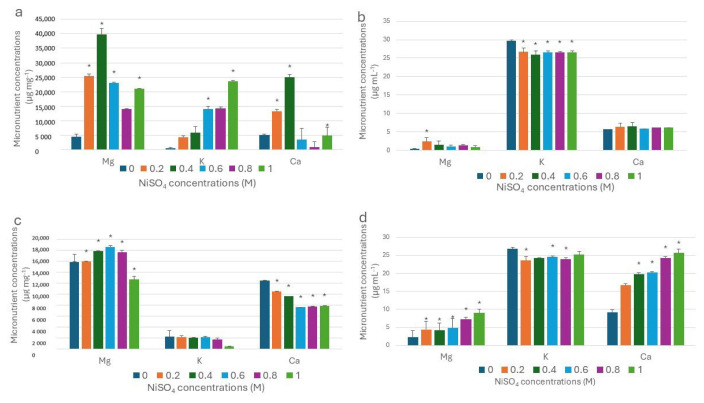
Uptake of Mg(II), K, and Ca(II) ions in **(a)** sample of Scenedesmus aldavei dried biomass, **(b)** sample of Scenedesmus aldavei supernatant, **(c)** sample of Desmodesmus pannonicus dried biomass, and **(d)** sample of Desmodesmus pannonicus supernatant under NiSO4 toxicity.

Changes of macro- and micronutrient uptake capacity in *Desmodesmus pannonicus* and *Scenedesmus aldavei* caused by metal concentrations, as measured from both biomass and supernatant, are presented in Figures 5 and 6.

The variations observed in macro- and microelement levels under Ni(II) exposure indicate that nickel stress strongly influences ionic balance and nutrient uptake mechanisms in both algal species. In *Scenedesmus aldavei*, Mg(II), K, and Zn(II) levels increased across all tested concentrations, whereas Ca(II) increased at 0.2 and 0.4 M NiSO_4_ but decreased at higher concentrations (0.6–1 M). Although Na^+^ levels increased under all treatments, the highest increase was detected at 0.2 M NiSO_4_, and the magnitude of this increase gradually declined at higher nickel concentrations. In contrast, in the supernatant samples, Na^+^ increased at all concentrations, Mg(II) displayed concentration-dependent changes, K^+^ decreased throughout all treatments, and Ca(II) showed significant reductions at 0.2–0.8 M NiSO_4_ (p ≤ 0.05).

A different response pattern was observed for *Desmodesmus pannonicus*. In the supernatant samples, Na^+^ levels decreased significantly according to ANOVA and Tukey post hoc test results. Within the algal biomass, Ca(II) decreased at all concentrations, while Mg(II) increased at 0.6 M NiSO_4_ but decreased at 0.8 and 1 M concentrations. This finding suggests that Ni(II) exposure may stimulate Mg(II) uptake up to a threshold concentration, whereas excessive nickel levels impair nutrient assimilation and ionic homeostasis. Similarly, all measured ion levels increased at 0.2–0.8 M NiSO_4_ but declined at 1 M, indicating that severe nickel stress disrupts mineral regulation mechanisms.

These findings are consistent with previous studies demonstrating that heavy metal uptake in microalgae is closely associated with ion exchange, surface complexation, adsorption, and microprecipitation processes. Mehta and Gaur (2005) reported that algal metal uptake mechanisms are strongly influenced by ion-exchange reactions and cell-surface complexation processes. In addition, competition among cations with similar valencies for negatively charged binding sites on the algal cell wall may alter the uptake efficiency of both essential nutrients and toxic metals. Rodea-Palomares et al. (2009) demonstrated that increased Ca(II) and Mg(II) concentrations reduced Zn(II) uptake in *Ulva reticulata* due to the formation of Ca/Mg carbonates, bicarbonates, and hydroxide precipitates during biosorption. Similarly, Senthilkumar et al. (2006) emphasized that the presence of competing cations can significantly reduce metal uptake due to competition for common adsorption sites.

The present findings also agree with the observations of Bucková et al. (2023), who investigated nutrient uptake mechanisms in *Desmodesmus subspicatus* exposed to K_2_Cr_2_O_7_, ZnCl_2_, and NiCl_2_·6H_2_O. Their study identified Ni(II) as the most toxic metal among the tested compounds and demonstrated that the presence of additional metals reduced Ni(II) removal efficiency. The decreases observed in Ca(II), K^+^, and Mg(II) levels at higher NiSO_4_ concentrations in the present study support the idea that nickel toxicity disrupts ionic equilibrium and interferes with the uptake of physiologically important elements. Furthermore, contrasting ion-regulation patterns observed between *Scenedesmus aldavei* and *Desmodesmus pannonicus* indicate species-specific differences in metal tolerance and nutrient management strategies under nickel stress. In particular, the ability of *S. aldavei* to maintain or increase the levels of various ions under Ni exposure may contribute to its comparatively higher nickel tolerance and biosorption capacity.

## 4. Conclusion

The present study has demonstrated that NiSO_4_ treatment significantly affected the growth dynamics, photosynthetic pigment content, nutrient uptake mechanisms, and biosorption capacities of *Desmodesmus pannonicus* and *Scenedesmus aldavei* over a 14-day exposure period. Growth curves, including OD_750_ absorbance and chlorophyll-*a* and chlorophyll-*b* levels, differed markedly from those of the control groups for both species, indicating that nickel stress directly influences photosynthetic activity and cellular growth. Although both species exhibited inhibitory responses at increasing NiSO_4_ concentrations, *Scenedesmus aldavei* maintained chlorophyll-*a* stability for a longer period and showed a considerably higher EC_50_ value than *Desmodesmus pannonicus*, demonstrating a stronger tolerance to nickel stress. In addition, both species exhibited bioremediation capability at concentrations of up to 1 M NiSO_4_; however, *S. aldavei* displayed a remarkably higher Ni(II) biosorption capacity than *D. pannonicus*. When evaluated alongside previous studies, these findings indicate that *Scenedesmus aldavei* is a highly promising model organism for phycoremediation and heavy-metal removal studies.

This study also revealed that Ni(II) exposure significantly altered macro- and micronutrient uptake patterns in both algal species. Variations in Na^+^, Mg(II), Ca(II), and K^+^ levels demonstrated that nickel stress disrupts ionic balance and nutrient regulation mechanisms in algal cells. In particular, Na^+^ uptake generally increased with NiSO_4_ application, whereas Mg(II), Ca(II), and K^+^ contents varied depending on nickel concentration and species-specific responses. FTIR analyses further indicated that functional groups including hydroxyl, carboxyl, amine, and polysaccharide-associated groups play important roles in nickel binding and biosorption. Overall, the results confirm that high nickel concentrations strongly influence both physiological activity and mineral uptake capacity in freshwater microalgae. They also demonstrate that *Scenedesmus aldavei* possesses exceptional resistance and biosorption potential that may be advantageous for future environmental bioremediation applications.

## Figures and Tables

**Table:** Assignments of absorbance bands in FTIR spectra.

Wavelengths (cm^−1^)	Functional group	*Desmodesmus pannonicus* (control)	*Scenedesmus aldavei* (control)	*Desmodesmus pannonicus* (NiSO_4_)	*Scenedesmus aldavei* (NiSO_4_)
3030–3700	Water stretching (-OH); protein stretching (-NH)	3277	3277	3345	3320
2820–3030	-CH_3_ asymmetric and -CH_3_ symmetric	2922–2851	2922–2851	2928–2854	2981–2919
1579–1715	Amid I protein band; stretching (C=O)	1638	1638	1631	1631
1484–1580	Amid II protein band	1536	1533	1505	1505
1271–1484	Amid III protein band	1360	1391	1388	1394
1132–1197	Polysaccharides carbohydrate (-COH); nucleic acid with stretching of phosphodiester (P=O).	1144	1150	1150	1150
980–1130	Polysaccharides carbohydrates (C-O-C and C-OH)	1023	1023	1039	1045
400–950	Outside the vibrational plane (CH)	826–557	813–572	832–594	832–591

## Data Availability

The data supporting the findings of this study are available from the corresponding author upon reasonable request.
